# A Systematic Histopathologic Evaluation of Type-A Aortic Dissections Implies a Uniform Multiple-Hit Causation

**DOI:** 10.3390/jcdd8020012

**Published:** 2021-01-27

**Authors:** Nimrat Grewal, Bart J. J. Velders, Adriana C. Gittenberger-de Groot, Robert Poelmann, Robert J. M. Klautz, Thomas J. Van Brakel, Jan H. N. Lindeman

**Affiliations:** 1 Department of Cardiothoracic Surgery, Leiden University Medical Center, 2333 ZA Leiden, The Netherlands; b.j.j.velders@lumc.nl (B.J.J.V.); r.j.m.klautz@lumc.nl (R.J.M.K.); t.j.van_brakel@lumc.nl (T.J.V.B.); 2 Department of Anatomy and Embryology, Leiden University Medical Center, 2333 ZA Leiden, The Netherlands; a.c.gitten@lumc.nl; 3 Department of Cardiology, Leiden University Medical Center, 2333 ZA Leiden, The Netherlands; 4 Institute of Biology, Animal Sciences and Health, Leiden University, 2333 BE Leiden, The Netherlands; R.E.Poelmann@lumc.nl; 5 Department of Vascular Surgery, Leiden University Medical Center, 2333 ZA Leiden, The Netherlands; J.H.N.lindeman@lumc.nl

**Keywords:** acute aortic syndrome, cardiovascular disease, aorta

## Abstract

(1) Background: The pathophysiologic basis of an acute type A aortic dissection (TAAD) is largely unknown. In an effort to evaluate vessel wall defects, we systematically studied aortic specimens in TAAD patients. (2) Methods: Ascending aortic wall specimens (*n* = 58, mean age 63 years) with TAAD were collected. Autopsy tissues (*n* = 17, mean age 63 years) served as controls. All sections were studied histopathologically. (3) Results: Pathomorphology in TAAD showed predominantly moderate elastic fiber fragmentation/loss, elastic fiber thinning, elastic fiber degeneration, mucoid extracellular matrix accumulation, smooth muscle cell nuclei loss, and overall medial degeneration. The control group showed significantly fewer signs of those histopathological features (none-mild, *p* = 0.00). It was concluded that the dissection plane consistently coincides with the vasa vasorum network, and that TAAD associates with a significantly thinner intimal layer *p* = 0.005). (4) Conclusions: On the basis of the systematic evaluation and the consistent presence of diffuse, pre-existing medial defects, we hypothesize that TAAD relates to a developmental defect of the ascending aorta and is caused by a triple-hit mechanism that involves (I) an intimal tear; and (II) a diseased media, which allows (III) propagation of the tear towards the plane of the vasa vasorum where the dissection further progresses.

## 1. Introduction

An acute aortic dissection is a medical catastrophe [1] with an estimated mortality of 26% in patients undergoing acute surgery and up to 58% in patients not receiving surgical treatment [2]. Reported incidences of an aortic dissection vary between 3 and 9 per 100,000 person-years [1,2,3,4], but with an aging population further increases are anticipated for the future.

An aortic dissection is hallmarked by a tear in the intimal layer, which allows blood to access the middle layer of the wall, causing the vascular layers to separate (dissect) from one another, resulting in formation of a ‘’false’’ lumen. One or multiple re-entry tears down- or upstream allow flow in the false lumen. Pressure build-up in the false lumen may result in compression of the aortic side branches. Depending on the degree of occlusion, and the side branches involved, this may result in life-threatening ischemia. 

The large majority of dissections are continuous with the actual arterial lumen via one or more intimal ‘’entry tears’’, yet some dissections may present without an apparent continuity with the actual lumen [1]. Based on their location and extent, an aortic dissection is classified as Stanford type-A (TAAD) dissections (dissections involving the aortic root and/or ascending aorta, which can propagate to the aortic arch and further into the descending aorta), and type-B dissections (dissections that do not involve the ascending aorta) [5]. The focus of this study is on TAAD.

The pathophysiologic basis of TAAD is poorly understood. Although the disease is associated with hypertension [1,2,3], the great majority of patients with hypertension will not develop a dissection. Observed associations between sporadic TAAD and genetic disorders that associate with structural aortic defects such as Marfan-, Ehlos-Danlos-, and Turner’s syndrome [1,3] imply impaired aortic wall integrity as a predisposing factor for TAAD. 

A role for (an) underlying aortic defect(s) as predisposing factor for TAAD is further supported by a number of histological evaluations [6,7,8]. Unfortunately, while diffuse medial defects are consistently reported in the literature, the published studies are extremely heterogeneous with respect to the histopathological descriptions and the nomenclature used [9]. Moreover, most evaluations so far are based on conventional Haematoxylin and Eosin (H&E) staining, a staining that while highly useful for obtaining a gross overview of cell shape and cellular density lacks the level of information required for the appreciation of putative matrix defects.

With the aim of harmonizing the histomorphological characterization of degenerative vascular pathologies, the Society for Cardiovascular Pathology along with the Association for European Cardiovascular Pathology recently issued a consensus statements with an unambiguous nomenclature [9]. This nomenclature allows for a systematic and uniform description of the histomorphology of non-inflammatory degenerative vascular pathologies [9]. In order to systematically map and describe putative aortic wall defects in TAAD, we now applied the consensus scoring and nomenclature on histological sections of 58 successive TAAD patients that underwent ascending aortic replacement for a type-A dissection. In order to optimally map putative matrix defects, H&E staining was combined with MOVAT pentachrome and Resorcin Fuchsin (elastin) stainings.

## 2. Materials and Methods

### 2.1. Ethics

Our study complies with the Declaration of Helsinki. Approval for this study was granted by the Medial Ethical Committee of Leiden University Medical Center (LUMC), Leiden. All control specimens were obtained post mortem. Obduction has been performed according the guidelines of the pathology department. Tissue collection was performed according to the regulations and protocols for secondary tissue use of the dept. of pathology at the Leiden University Medical Center. We have obtained permission to include the specimens in our study and obduction. 

### 2.2. Patients and Tissue Samples

Ascending aortic wall samples were obtained from the aortotomy site as residual aortic wall material during an ascending aortic replacement in patients with acute TAAD. Seventeen control aortas were obtained during post-mortem autopsies. The wall samples were formalin-fixed, decalcified using Kristensens solution, and paraffin-embedded. 

Successive 4 µm sections were, respectively, Haematoxylin-Eosin (HE)-, Resorcin-Fuchsin (RF)-, and MOVAT-pentachrome-stained. Vasa vasorum were visualized by CD31 staining (M0823, Agilent, Amsterdam, the Netherlands), 1/1000 after Tris EDTA (0.01 M, pH 8) heat retrieval. The anti-mouse Envision+ System HRP (K4007, Agilent) and DAB solution (K3468, Agilent) were used for visualization.

### 2.3. Morphology

Histomorphological grading was performed according to the consensus classification for degenerative aorta pathology [9]. Elastic fiber fragmentation/loss, elastic fiber thinning, elastic fiber disorganisation, mucoid extracellular matrix accumulation, smooth muscle cell nuclei loss, and overall medial degeneration were described as none (0), mild (1), moderate (2), or severe (3). Standard H&E staining was used to examine smooth muscle cell nuclei loss; elastic fiber fragmentation/loss, elastic fiber thinning, and elastic fiber disorganisation were graded by RF staining, and mucoid extracellular matrix accumulation and medial degeneration were scored in the MOVAT pentachrome staining. Scoring was performed by two independent researchers. Inter-observer variation was estimated with Cohen’s kappa coefficient [10]. Excellent (>0.8) Cohen’s kappas [10] were observed for all parameters (Table 1).

### 2.4. Anatomical Location of Dissection Plane

**Figure 1:**
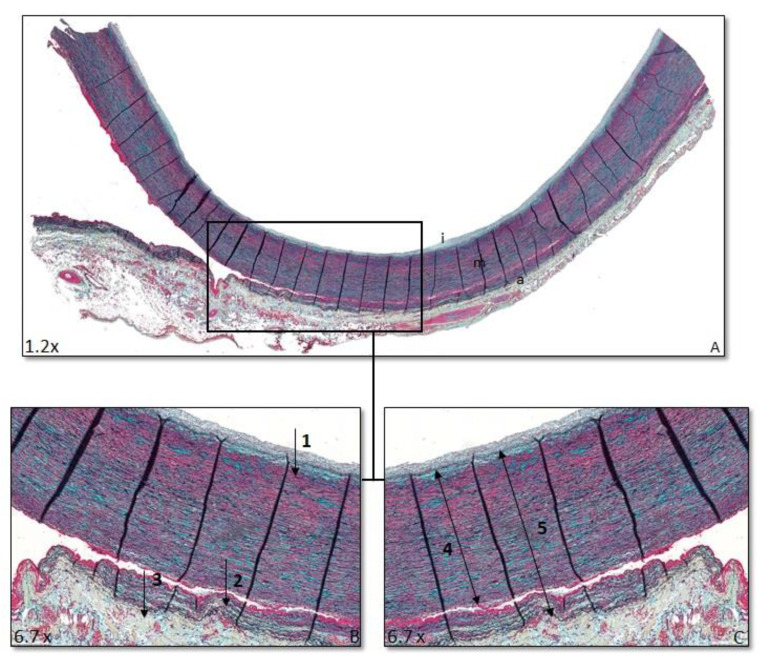
Ratio dissection plane. Transverse histologic sections of the ascending aorta 4 µm stained with MOVAT pentachrome staining. (**A**) Overview ascending aorta I = intima, m = media, a = adventitia. (**B**) 1: Lamina elastica interna (LEI), 2: dissection plane (DP), and 3: lamina elastica externa (LEE). (**C**) 4: Distance between LEI and DP, 5: distance between LEI and LEE. Ratio dissection plane is defined as distance 4 divided by distance 5.

**Figure 2:**
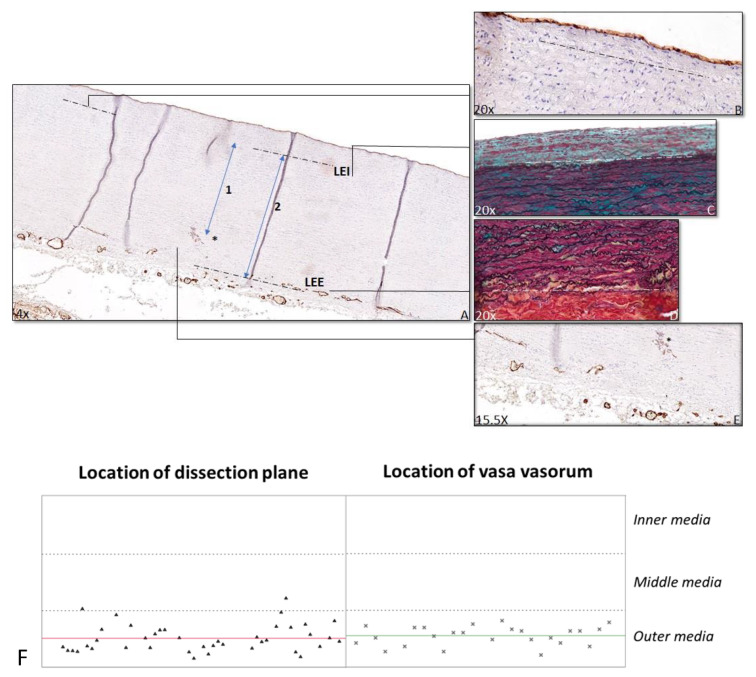
Ratio vasa vasorum. Transverse histologic sections 4 μm stained for CD31 (**A**,**B**,**E**) and MOVAT (**C**,**D**) in a control patient (**A**–**E**). **A** shows an overview of the ascending aorta, with the dashed lines indicating the lamina elastica interna (LEI), with a detailed view of the LEI in **B** in a CD31 stained section and in **C** in a MOVAT stained section. The second dashed line (**A**) indicates the lamina elastica externa (LEE), with a detailed view in the MOVAT stained section (**D**). The asterisk indicates the nearest vasa vasorum (**A**,**E**). The ratio of the vasa vasorum is calculated by dividing the distance between LEI and nearest vasa vasorum (indicated by 1 in **A**) by the distance between LEI and LEE (indicated by 2 in **A**). The location of the dissection plane is shown on the left and the vasa vasorum on the right in graph **F**. Each triangle and asterisk (**F**) represents one TAAD patient. The red line represents the median location of the dissection plane, and the green line represents the median location of vasa vasorum. The dashed lines are the borders between the inner, middle, and outer media.

The relative position of the dissection plane in the media was estimated as the ratio of the distance from the first major internal elastic lamella (lamina elastica interna) to the dissection plane over the full medial thickness (i.e., inner to outer elastic lamina (lamina elastica externa) (Figure 1). For the relative position of the vasa vasorum, the ratio of the distance from the lamina elastica interna to the vasa vasorum and the full medial thickness were calculated (Figure 2). Distance ratios were assessed for three different fields using Philips Standalone Viewer software (Philips, Eindhoven, The Netherlands), and the average relative position was used. 

### 2.5. Statistical Analysis

Statistical analysis was performed using the IBM SPSS statistics software, version 26. Chi-square test was used to compare the morphology between the dissection and the control group. Independent samples *t*-test was used to compare the ratio of the dissection plane to ratio of the vasa vasorum plexus.

## 3. Results

### 3.1. Patient Characteristics 

This study includes 58 successive TAAD patients and 17 control patients. Baseline characteristics of the patients are shown in Table 2. 

### 3.2. Histopathology of the Vascular Wall

**Figure 3:**
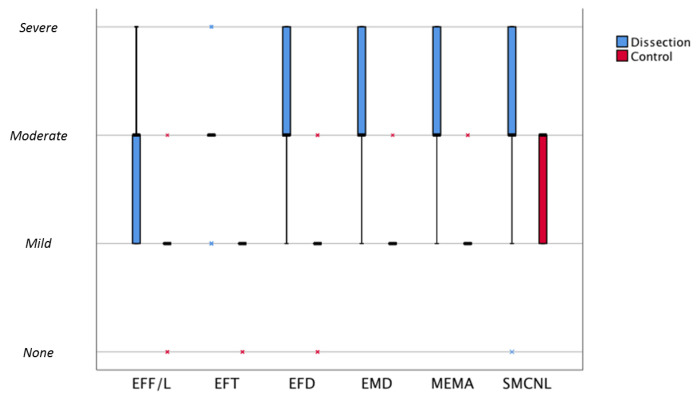
Graph of median morphology scores comparing the dissection and control group. Boxplot of median morphological scores comparing the dissection and control group. Boxes represent median morphological score (bold horizontal lines) and interquartile ranges (75%; 25%). Extremes are presented as asterisks. Blue corresponds to the dissection group and red to the control group. EFF/L: elastic fiber fragmentation/loss; EFT: elastic fiber thinning; EFD: elastic fiber degeneration; OMD: overall medial degeneration; MEMA: mucoid extracellular matrix accumulation; SMCNL: smooth muscle cell nuclei loss.

Putative aortic wall abnormalities in TAAD were mapped according to the revised, consensus nomenclature for noninflammatory, degenerative aortic histopathologies [9]. Conclusions for this systematic evaluation of TAAD wall and control aorta are summarized in Table 3 and Figure 3. While all degenerative aspects covered in the consensus classification were classified moderately severe in TAAD, in the control group predominantly mild degenerative aspects were observed, with a significant difference between both groups (Table 3) (Figure 3). After excluding the BAV and MFS patients from the TAAD group, the observed differences remained significant. 

**Figure 4:**
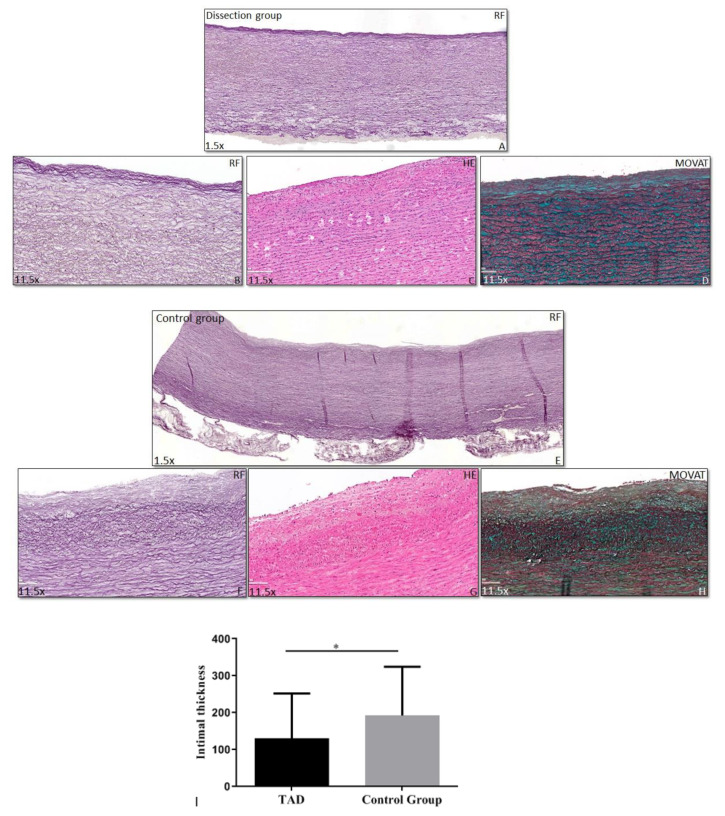
Intimal thickness. Transverse histologic sections (4 μm) stained for Resorcin Fuchsin (RF), Haematoxylin Eosin (HE), and MOVAT. The intimal layer, indicated with the dashed line, is significantly thinner in the dissection group (a patient without Marfan or a bicuspid aortic valve) (**A**–**D**) than in the control group (**E**–**H**) (Graph **I**). Magnification (**A**,**E**) 1.5×, (**B**–**D**,**F**–**H**) 11.5×, *: *p* < 0.05.

Wall thicknesses in TAAD and control were similar, but the intimal thickness was consistently less in the TAAD group (mean thickness 131 ± 122 μm (mean ± sd) vs. 193 ± 132 μm (mean ± sd) in the control group (*p* = 0.044) (Figure 4). Intimal thickness of the 6 patients with Marfan syndrome or BAV disease included in this study was 47 ± 29 μm (mean ± sd). After excluding the BAV and MFS patients from the TAAD group, the intima was still significantly thinner than in the control group. 

### 3.3. Dissection Plane

From the histological evaluation, the picture emerged of a uniform dissection plane for all TAAD specimens in the study. We therefore evaluated the level of the dissection plane for the 41 cases for which full-thickness aortic wall samples were available. For the remaining 17 samples, the adventitial aspect of the media was either lost or fully separated from the media in the process of tissue procurement. In the available full thickness samples, the dissection plane was consistently located in the outer aspect of the media (at 83% (SD 0.08) of the full medial thickness), a level that coincides with the aortic vasa vasorum network (Figure 2). The level of the vasa vasorum was calculated in the specimen with an intact vasa vasorum network in the outer media (*n* = 25).

## 4. Discussion

This systematic histological evaluation of aorta segments of type A dissections shows universal presence of diffuse, moderately-severe medial defects, a thinner intima, and a dissection plane that is consistently located at the level of the vasa vasorum network in the outer media. On basis of these observations, the picture emerges of aortic dissection being the consequence of a multi-hit process that involves a potentially more vulnerable (thinner) intima, a pre-existing incompetent media that is unable to withstand the tear forces exerted by the blood following an intimal tear, thereby allowing the blood access to an area of least resistance (the actual dissection plane) at the level of the vasa vasorum network in the outer medial layer.

Histological evaluation indicated universal presence of medial defects in all TAAD cases included in this evaluation. Medial defects were systematically inventoried by applying the recently issued consensus grading from the Society for Cardiovascular Pathology and the Association For European Cardiovascular Pathology [9]. This consensus grading system was developed in order to harmonize the nomenclature for histopathology of vascular diseases. The ultimate aim of the nomenclature is to facilitate data integration of results obtained from different groups or from different diseases [11]. Evaluation of the grading performance showed excellent Cohen’s kappas for all parameters in the consensus grading.

This is the first study to apply the consensus classification to TAAD wall samples. Based on this grading, it was concluded that TAAD universally associates with moderately severe elastic fiber pathology, overall medial degeneration, mucoid extracellular matrix accumulation, and smooth muscle cell nuclei loss. Additionally, MOVAT staining implied further pathological changes not included in the consensus classification [11] such as extensive (micro) fibrotic changes [12] and adventitial remodeling in subset of cases.

Apart from the consistent presence of the moderately severe medial defects, this systematic histological inventory also revealed a remarkably thin intima, and the existence of uniform dissection plane, as consistent findings in the 58 TAAD cases.

Although one could speculate that the observed thin intima in TAAD constitutes the primary defect, with thin intima’s being more prone to dissection and the medial defects reflecting a bystander phenomenon, such a theory is not compliant with the clinical observations that intimal tears or medial injuries in a healthy vessel do not result in dissection. Localized intimal tears are observed as incidental findings in elderly patients or after trauma [13]. Similarly, iatrogenic medial injuries following aortic cannulation [14], subintimal angioplasty [15], and end-atherectomy [16] do not associate clinically with dissection. Consequently, a healthy, competent media is able to fully resist exposure to perpendicular dissecting forces that develop when directly exposed to the blood stream. Mechanistically, this stability resides in the tissue’s network behavior that allows forces to be evenly distributed over the wall, and that prevents dissection of the individual layers [17,18].

An alternative explanation for the thin intima is that it reflects an underlying developmental and/or differentiation defect of the mesenchymal cell population [19]. Relative thin intima’s are present in Marfan Syndrome and in aortas from patients with bicuspid valve disease [20,21], conditions with clear developmental aortic maturation defects [22]. The universal finding of a reduced intimal thickness and the diffuse, moderately severe medial pathology suggest that TAAD relates to some form of underlying developmental defect in the ascending aorta.

A further remarkable observation was the uniform localization of the dissection plane (a preferential position in the outer media was reported in two earlier reports) [23,24]. The more detailed analysis herein shows that the dissection plane consistently co-localizes with the plane of the vasa vasorum network in the outer media.

One could speculate that the level harboring this vascular network structure is an inherently mechanically weak plane that is less able to resist the wedge forces exhibited by the blood than the other segments of the media, thereby creating an inherently weak plane within the aortic wall along which the dissection progresses. Considering the fact that the mean blood pressure in the vasa vasorum network is lower than the central blood pressure, we consider it unlikely that TAADs that present without an apparent continuity with the actual lumen relate to a bleeding from the vasa vasorum plexus.

In conclusion: this systematic histological evaluation of 58 successive TAAD shows that TAAD uniformly associates with pre-existing ascending aortic wall defects and consistently proceeds along a plane that coincides with the vascular plexus in the outer media. On this basis, a picture emerges of a TAAD-susceptible phenotype that relates to an inherently mechanically incompetent media, and a common final pathway reflecting propagation of the dissection along the plane of least resistance (the vasa vasorum network).

This is an observational study. As such, the study comes with several limitations: the evaluation is based on histological evaluation surgical specimens obtained at the time of aorta replacement, hence information on non-operated patients is missing. Moreover, information on the natural history of the medial changes, and on the molecular aspects of TAAD, is missing.

## Figures and Tables

**Table 1:** Morphological aspects included in the Pathology Consensus Score with the calculated kappa coefficient.

Morphological Feature	Kappa
Elastic fiber fragmentation/Loss	0.915
Elastic fiber thinning	0.880
Elastic fiber degeneration	0.801
Mucoid extra cellular matrix accumulation	0.938
Smooth muscle cell nuclei loss	0.978
Overall medial degeneration	0.879

**Table 2:** Baseline characteristics.

Variable	Type A Aortic Dissection Group *n* = 58	Control Group *n* = 17
Age, years	63 ± 10.36	63 ± 5.46
Gender		
- Female - Male - Unknown	- 26 (45%) - 31 (53%) - 1 (2%)	- 9 (53%) - 8 (47%) - 0 (0%)
Arterial hypertension		
- Yes - No - Unknown	- 28 (48%) - 29 (50%) - 1 (2%)	- 5 (29%) - 5 (29%) - 7 (42%)
Peripheral arterial disease		
- Yes - No - Unknown	- 0 (0%) - 57 (98%) - 1 (2%)	- 0 (0%) - 0 (0%) - 17 (100%)
Valve Morphology		
- Tricuspid aortic valve - Bicuspid aortic valve - Unknown	- 52 (90%) - 4 (7%) - 2 (3%)	- 17 (100%) - 0 (0%) - 0 (0%)
Connective tissue disorder		
- Marfan syndrome	- 2 (4%)	- 0 (0%)
Diabetes Mellitus		
- Yes - No - Unknown	- 1 (2%) - 57 (98%) - 0 (0%)	- 1 (6%) - 9 (53%) - 7 (41%)

**Table 3:** Histomorphological features in the aortic dissection and control group.

Morphological Feature	Score	Type A Aortic Dissection Group N (%)	Control Group N (%)	*p*-Value
Elastic fiber fragmentation/loss	- None - Mild - Moderate - Severe	- 0 (0) - 19 (33) - 29 (50) - 10 (17)	- 2 (12) - 14 (82) - 1 (6) - 0 (0)	0.000
Elastic fiber thinning	- None - Mild - Moderate - Severe	- 0 (0) - 12 (21) - 41 (71) - 5 (8)	- 2 (12) - 15 (88) - 0 (0) - 0 (0)	0.000
Elastic fiber disorganization	- None - Mild - Moderate - Severe	- 0 (0) - 13 (23) - 28 (48) - 17 (29)	- 2 (12) - 13 (76) - 2 (12) - 0 (0)	0.000
Overall medial degeneration	- None - Mild - Moderate - Severe	- 0 (0) - 6 (10) - 27 (47) - 25 (43)	- 0 (0) - 16 (94) - 1 (6) - 0 (0)	0.000
Mucoid extracellular matrix accumulation	- None - Mild - Moderate - Severe	- 0 (0) - 8 (10) - 32 (55) - 18 (31)	- 0 (0) - 15 (88) - 2 (12) - 0 (0)	0.000
Smooth muscle cell nuclei loss	- None - Mild - Moderate - Severe	- 0 (0) - 10 (17) - 33 (57) - 14 (24)	- 0 (0) - 8 (47) - 9 (53) - 0 (0)	0.023

## Data Availability

The data presented in this study are available on request from the corresponding author. The data are not publicly available due to ethical reasons.
